# Compartment-specific roles of gasdermin D and E in photoreceptor degeneration: evidence and pharmacological implications

**DOI:** 10.3389/fphar.2026.1981060

**Published:** 2026-10-29

**Authors:** Ying Deng, Yasha Zhou, Pai Zhou, Li Xiao, Jing Lu, Qinghua Peng, Yijing Yang

**Affiliations:** 1 Hunan University of Chinese Medicine, Changsha, China; 2 Institute of Ophthalmology and Otolaryngology of Chinese Medicine, Changsha, China; 3 Changsha Centre for Innovation in Traditional Chinese Medicine Techniques for the Prevention and Treatment of Retinal Diseases and Visual Function Protection, Changsha, China

**Keywords:** ferroptosis, gasdermin D, gasdermin E, inflammasome, photoreceptor degeneration, pyroptosis, retinal neuroinflammation

## Abstract

Photoreceptor degeneration arises from diverse genetic and acquired insults, yet oxidative stress, mitochondrial dysfunction, and sterile inflammation repeatedly converge on membrane injury and irreversible cell loss. Inflammasome activation is frequently described as retinal pyroptosis, although upstream markers alone neither establish gasdermin-dependent lysis nor identify the executing cell. Rather than treating retinal pyroptosis as a single cell-autonomous pathway, we distinguish a myeloid gasdermin D (GSDMD) inflammatory relay from a photoreceptor-intrinsic gasdermin E (GSDME) lytic amplification program and evaluate potential interventions according to direct retinal evidence, cellular specificity, and translational readiness. GSDMD is best supported in microglia and subretinal macrophages, where inflammasome-caspase-1 signaling promotes cytokine and extracellular-vesicle release and can worsen secondary photoreceptor injury. Recent evidence in TREM2-deficient rd10 retina further supports GSDMD as a functional disease modifier, while remaining context specific. By contrast, GSDME has direct loss-of-function support in all-trans-retinal overload, in which caspase-3-dependent cleavage increases membrane rupture and is associated with mitochondrial oxidative burden and ferroptotic susceptibility. We compare NLRP3/caspase-1, interleukin-1, GSDMD pore, GSDME, and redox-directed strategies against five translational gates: target engagement, ocular exposure, cell specificity, delivery feasibility, and safety. This evidence-graded framework identifies promising pharmacological entry points while preserving the distinction between mechanistic plausibility and retinal target readiness.

## 1 Introduction

Inherited retinal degenerations, including retinitis pigmentosa, comprise genetically heterogeneous disorders in which primary rod injury is frequently followed by secondary cone dysfunction and death. Acquired conditions such as retinal detachment, photo-oxidative damage, Stargardt disease, and age-related macular degeneration likewise converge on photoreceptor stress. Across these settings, high oxygen consumption, abundant polyunsaturated membrane lipids, continuous retinoid turnover, and close communication with retinal pigment epithelium and resident immune cells create a tissue that is unusually sensitive to oxidative and inflammatory amplification. Genetic ablation of caspase-1 protected photoreceptors in an autosomal dominant retinitis pigmentosa model, and NLRP3 deletion preserved bystander cones in P23H rhodopsin degeneration, indicating that inflammasome-related signaling can modify inherited disease progression (Samardzija et al., 2006; Viringipurampeer et al., 2016).

These observations have often been summarized under the label 'pyroptosis’. That label is mechanistically demanding. Pyroptosis is not established by increased NLRP3, caspase-1, interleukin-1β (IL-1β), or interleukin-18 (IL-18) alone; it requires a pore-forming gasdermin and evidence that membrane permeabilization depends on that effector. The distinction matters in retina because inflammasome activation may occur in microglia while photoreceptors die secondarily, whereas a different gasdermin may act inside the photoreceptor. A cell-resolved view therefore offers more explanatory and therapeutic value than treating retinal pyroptosis as a single linear pathway.

Previous reviews have summarized inflammasome activation and pyroptosis across retinal diseases or gasdermin biology across neurodegenerative disorders (Zhao et al., 2022; Sun et al., 2024; Chen et al., 2026). However, the distinction between gasdermin executor identity, cellular ownership, and pharmacological target readiness remains insufficiently resolved in photoreceptor degeneration. Here, we therefore separate a predominantly myeloid GSDMD inflammatory relay from photoreceptor-intrinsic GSDME-mediated lytic amplification and evaluate each pathway according to causal evidence and translational maturity. This compartment-based comparison, rather than a catalogue of retinal inflammasome markers, is the central contribution of the review.

This article is a focused narrative review rather than a systematic review or meta-analysis. Targeted searches of PubMed and Web of Science were conducted for English-language literature available through August 2026 using combinations of gasdermin, pyroptosis, photoreceptor, retinal degeneration, microglia, inflammasome, and ferroptosis; reference lists of key retinal studies and relevant reviews were also screened. We prioritized mechanistic retinal studies reporting active-fragment localization, membrane permeabilization, gasdermin loss of function or rescue, pharmacological intervention, and retinal structural or functional outcomes. Representative extra-retinal studies were included only to define gasdermin biology or clearly label testable hypotheses. Owing to heterogeneity in models and endpoints, study selection was interpretive and no pooled effect sizes or formal risk-of-bias assessment were performed.

## 2 Gasdermin pores: an endpoint shared by distinct upstream programs

Gasdermins contain an N-terminal pore-forming domain restrained by a C-terminal autoinhibitory domain. In canonical inflammasome signaling, caspase-1 cleaves GSDMD; in noncanonical signaling, human caspase-4/5 or murine caspase-11 performs the corresponding step. The liberated GSDMD-N fragment binds acidic phospholipids, oligomerizes, and forms membrane pores that permit ion flux and cytokine release (Shi et al., 2015; Kayagaki et al., 2015; Sborgi et al., 2016). Pore formation does not invariably cause immediate lysis. Living cells may release IL-1 family cytokines through sublytic GSDMD pores, and calcium-triggered ESCRT recruitment can repair damaged membrane (Evavold et al., 2018; Ruhl et al., 2018). Thus, cleavage, cytokine secretion, pore formation, and terminal cell death are related but separable outcomes.

GSDME is activated principally by apoptotic caspase-3. In GSDME-expressing cells, this converts an initially non-lytic apoptotic program into rapid membrane permeabilization and secondary necrotic/pyroptotic morphology (Wang et al., 2017; Rogers et al., 2017). GSDME-N can also permeabilize mitochondria, promote cytochrome-c release, and reinforce caspase-3 activation, creating a feed-forward loop (Rogers et al., 2019). Consequently, the boundary between apoptosis and pyroptosis is determined partly by GSDME abundance, cleavage kinetics, membrane repair capacity, and the magnitude of stress(Figure 1).

**FIGURE 1:**
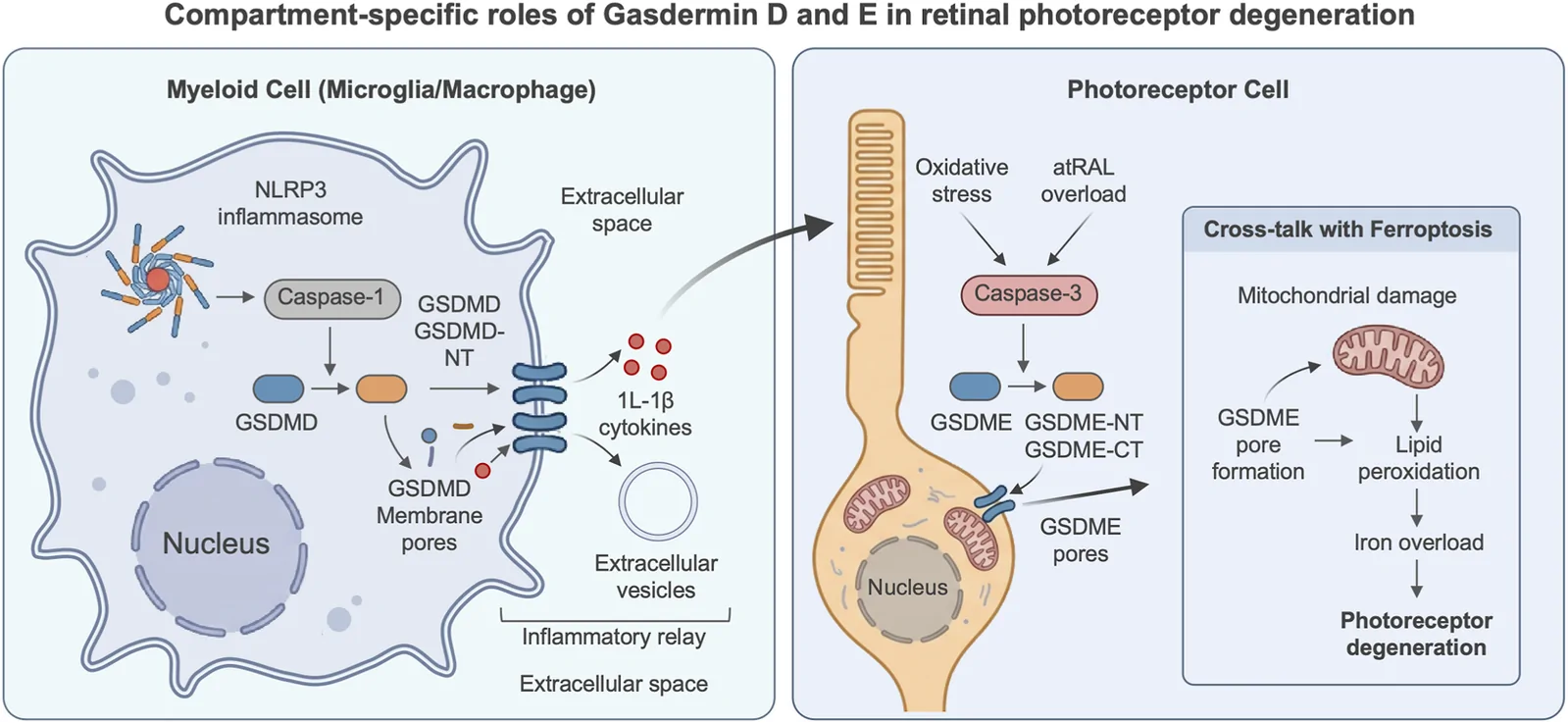
Compartment-specific gasdermin signaling in photoreceptor degeneration: established and proposed relationships. Left panel: In retinal myeloid cells (microglia/macrophages), NLRP3-caspase-1 signaling cleaves GSDMD, and GSDMD-N pore formation supports mature IL-1β and extracellular-vesicle release, generating an inflammatory relay that can worsen secondary photoreceptor injury. Right panel: In the atRAL-overload paradigm, oxidative stress activates caspase-3, produces GSDME-N, and promotes photoreceptor plasma-membrane permeabilization. Gsdme deletion also reduces mitochondrial ROS, lipid peroxidation, and iron accumulation; however, direct mitochondrial GSDME-N pore formation has not been demonstrated as the causal link to ferroptotic injury in photoreceptors. Solid arrows denote relationships supported by direct retinal evidence in the indicated compartment. Dashed arrows and question marks denote proposed, associative, or incompletely established relationships, particularly GSDME-mediated mitochondrial permeabilization and its connection to ferroptotic injury. Abbreviations: atRAL, all-trans-retinal; GSDMD/E, gasdermin D/E; IL-1β, interleukin-1β; ROS, reactive oxygen species.

For retinal studies, we assessed executor evidence against four criteria: (1) cell-type-resolved localization of the active N-terminal fragment; (2) membrane permeabilization or lytic morphology, assessed by propidium iodide uptake, lactate dehydrogenase release, membrane-pore imaging, or comparable assays; (3) gasdermin loss of function using a pore-impaired mutant, knockout, knockdown, or cleavage-resistant construct; and (4) rescue of retinal structure or function attributable to the same executor. The evidence-maturity terms in Table 1 are linked to these criteria. ‘Upstream retinal evidence’ denotes modification of a retinal phenotype by inflammasome or caspase manipulation without identification of the gasdermin executor. ‘Intermediate evidence’ includes active-fragment detection or a protective intervention but lacks definitive cell assignment and/or executor dependency. ‘Direct executor evidence’ requires cell-resolved fragment evidence plus gasdermin-specific loss of function and membrane or retinal rescue. ‘Strongest direct evidence’ indicates that all four criteria are substantially met within the same photoreceptor-resolved disease paradigm. Context-specific and generalizability qualifiers are retained even when direct evidence is strong.

**TABLE 1:** Evidence maturity and selected translational gates for gasdermin-related photoreceptor degeneration.

Model/compartment	Executor evidence	Tested intervention and outcome	Evidence maturity/limitation	Selected translational gates (retinal target engagement/ocular exposure/functional efficacy)
P23H/inherited RP	NLRP3 and caspase-1 modify degeneration, but the gasdermin executor and dying compartment were not established	Nlrp3 deficiency preserved bystander cones; caspase-1 ablation was protective in an RP model (Viringipurampeer et al., 2016; Samardzija et al., 2006)	Upstream retinal evidence; none of the four executor criteria is sufficient for direct gasdermin proof	Target engagement: no gasdermin-specific evidence. Ocular exposure: not applicable to genetic studies. Functional efficacy: retinal protection reported
Photo-oxidative injury/subretinal myeloid cells	GSDMD-positive cells were mainly IBA1-positive microglia/macrophages; a pore-impaired Gsdmd allele preserved structure and function	GSDMD siRNA reduced IL-1β release; Gsdmd I105N reduced inflammatory injury (Sekar et al., 2023)	Direct executor evidence: criteria 1, 3, and 4 are met, with pore-impaired genetics supporting criterion 2; knockout results suggest compensation	Target engagement: supported genetically and by reduced IL-1β release. Ocular exposure: not established. Functional efficacy: structural and retinal functional benefit reported
Retinal detachment/mixed compartments	GSDMD cleavage was detected, but later localization placed most early GSDMD-N in microglia rather than photoreceptors	GSDMD-C delivery and the caspase-1 inhibitor VX-765 reduced injury (Li et al., 2020; Cao et al., 2024)	Intermediate evidence: criteria 1 and 4 are partly met, but intervention cell specificity, direct pore dependency, and timing remain unresolved	Target engagement: partial (cleavage/inflammatory signaling). Ocular exposure: retinal delivery occurred, but exposure-response was not quantified. Functional efficacy: ONL preservation reported
TREM2-deficient rd10/myeloid-inflammatory context	Cleaved caspase-1 and GSDMD increased in retinal myeloid cells; global and Cx3cr1-directed Gsdmd loss mitigated photoreceptor loss	Disulfiram reduced GSDMD oligomerization and degeneration (Wang et al., 2026)	Direct, context-specific executor evidence: criteria 1, 3, and 4 are met and oligomerization supports criterion 2; not yet generalized to ordinary rd10	Target engagement: yes, reduced retinal GSDMD oligomerization. Ocular exposure: exposure-response not established. Functional efficacy: photoreceptor preservation reported in the sensitized model
atRAL overload/photoreceptors	Caspase-3-dependent GSDME-N localized to the outer nuclear layer; Gsdme deletion reduced rupture and degeneration	N-acetylcysteine reduced caspase-3/GSDME activation (Cai et al., 2022)	Strongest direct photoreceptor-intrinsic evidence: all four criteria are substantially met, but evidence derives from one principal disease paradigm	Target engagement: genetic dependency and reduced GSDME activation reported. Ocular exposure: not established for N-acetylcysteine. Functional efficacy: structural rescue; visual functional validation remains limited
atRAL-associated ferroptotic injury/photoreceptors	Gsdme deletion reduced mitoROS, iron loading, and lipid peroxidation, but direct mitochondrial GSDME-N pore causality was not shown	MitoTEMPO and JNK inhibition reduced oxidative or ferroptotic injury (Yang et al., 2025a; b)	Direct genetic association with incomplete mechanism: criteria 3 and 4 are supported, whereas mitochondrial localization/permeabilization criteria 1 and 2 remain unproven	Target engagement: not shown for GSDME by the pharmacological agents. Ocular exposure: not reported. Functional efficacy: biochemical and structural benefit reported; gasdermin-dependent treatment interaction is unresolved

Evidence maturity is assigned using the four criteria defined in the main text: active-fragment localization, membrane permeabilization, gasdermin loss of function, and structural or functional rescue. The selected translational-gate column reports retinal target engagement, ocular exposure, and functional efficacy; ‘not established’ indicates that the relevant study did not provide sufficient evidence. Abbreviations: atRAL, all-trans-retinal; GSDMD/E, gasdermin D/E; IL-1β, interleukin-1β; mitoROS, mitochondrial reactive oxygen species; ONL, outer nuclear layer; RP, retinitis pigmentosa.

## 3 GSDMD: an inflammatory relay with unresolved cellular ownership

Photo-oxidative retinal damage provides the strongest evidence that caspase-1-dependent inflammasomes contribute to focal photoreceptor degeneration. Genetic caspase-1 deficiency and pharmacological caspase-1 inhibition reduced inflammatory mediators and photoreceptor loss in this model (Wooff et al., 2020). Sekar and colleagues subsequently interrogated the terminal effector. Mice carrying the pore-impaired Gsdmd I105N variant showed better retinal function, greater photoreceptor survival, and lower inflammatory gene expression after photo-oxidative injury. Importantly, GSDMD-positive cells in the subretinal space were predominantly IBA1-positive microglia/macrophages. GSDMD also supported release of IL-1β-containing extracellular vesicles, suggesting that its major retinal function may be propagation of inflammation rather than direct execution of photoreceptors (Sekar et al., 2023).

The same study exposed an instructive complication: complete Gsdmd knockout did not reproduce all protection observed with the pore-impaired allele. Long-term absence of GSDMD may permit compensatory cytokine release or engagement of other gasdermins, whereas a functional pore mutation may interrupt injury signaling without producing the same developmental adaptation. This difference argues against treating knockout, cleavage inhibition, and pore blockade as interchangeable manipulations. It also supports testing acute, cell-restricted interventions in addition to germline loss.

Retinal detachment illustrates the remaining cell-identity controversy. One study detected GSDMD cleavage and reported that subretinal delivery of the inhibitory GSDMD-C domain reduced inflammatory signaling and photoreceptor degeneration, which was interpreted as compatible with photoreceptor pyroptosis, although the cellular specificity of the intervention was not directly established (Li et al., 2020). A later cell-localization study found that most early GSDMD-N-positive cells after detachment were IBA1-or P2RY12-positive microglia; the caspase-1 inhibitor VX-765 suppressed microglial pyroptosis and indirectly preserved the outer nuclear layer (Cao et al., 2024). These results need not be mutually exclusive, because the dominant GSDMD-expressing compartment may shift with time and injury severity. Nevertheless, they show why whole-retina immunoblotting cannot assign pyroptosis to photoreceptors.

A 2026 study in the Pde6b rd10 model adds the strongest inherited-disease evidence for GSDMD while also sharpening the compartment question. Global TREM2 deficiency increased inflammasome-associated signaling in retinal myeloid cells, including cleaved caspase-1, cleaved GSDMD, and mature IL-1β. Global or Cx3cr1-directed Gsdmd loss, and disulfiram-mediated pore inhibition, mitigated photoreceptor loss in the TREM2-deficient rd10 background (Wang et al., 2026). This establishes a functional GSDMD contribution in a genetically sensitized inherited model and supports myeloid ownership. It does not yet show that GSDMD is required in ordinary rd10 degeneration, nor that the executor acts within photoreceptors.

## 4 GSDME: a photoreceptor-intrinsic amplifier linking apoptosis and ferroptosis

Evidence for photoreceptor-intrinsic GSDME is more direct in disorders of all-trans-retinal (atRAL) clearance. In light-exposed Abca4-null/Rdh8-null mice and atRAL-loaded 661W cells, atRAL induced reactive oxygen species, JNK activation, mitochondrial disruption, caspase-3 cleavage, and GSDME-N formation. GSDME-N localized to the photoreceptor outer nuclear layer, and Gsdme deletion reduced membrane rupture, caspase-3 activation, and photoreceptor degeneration. N-acetylcysteine suppressed the caspase-3-GSDME response, positioning oxidative stress upstream of this lytic conversion (Cai et al., 2022).

A 2025 study extended this model beyond an apoptosis-to-pyroptosis switch. Gsdme deletion reduced mitochondrial reactive oxygen species, iron accumulation, and lipid peroxidation after atRAL overload, while the mitochondrial antioxidant MitoTEMPO attenuated photoreceptor ferroptosis and retinal atrophy (Yang et al., 2025b). Together with earlier evidence that defective atRAL clearance can drive ferroptosis (Chen et al., 2021), these findings support a GSDME-dependent increase in mitochondrial oxidative burden and ferroptotic susceptibility, but do not yet establish that mitochondrial GSDME-N pore formation is the direct causal step in photoreceptors. Death-pathway labels should therefore describe interacting molecular modules rather than mutually exclusive cell fates.

Generalization requires caution. The decisive GSDME studies use a shared atRAL-clearance paradigm, and comparable photoreceptor-specific loss-of-function data are not yet available across inherited models such as Pde6b-related rd10 degeneration. Moreover, reduced oxidative and ferroptotic readouts after Gsdme deletion could reflect less plasma-membrane rupture, altered mitochondrial stress, or secondary differences in cell survival. Distinguishing these possibilities will require compartment-resolved GSDME-N localization, direct pore or membrane assays, temporally controlled deletion, and rescue with cleavage-resistant or compartment-targeted constructs.

## 5 Pharmacological opportunities and translational constraints

Upstream inflammasome inhibition is the most pharmacologically mature entry point, but its retinal evidence is not equivalent to direct gasdermin target validation. The NLRP3 inhibitor MCC950 is well characterized in systemic inflammatory models (Coll et al., 2015), whereas genetic Nlrp3 deficiency and caspase-1 blockade provide retinal protection in P23H, photo-oxidative, or detachment paradigms (Viringipurampeer et al., 2016; Wooff et al., 2020; Cao et al., 2024). These interventions can reduce cytokine maturation and GSDMD cleavage simultaneously, yet they do not establish which gasdermin executes membrane injury. They may also suppress beneficial inflammasome functions, including antimicrobial defense and regulated cytokine release from viable cells.

Interleukin-1 pathway modulation is mechanistically narrower but remains upstream of lytic execution. An allosteric IL-1 receptor modulator reduced inflammation and photoreceptor toxicity in experimental retinal degeneration (Dabouz et al., 2020). Such protection supports an inflammatory contribution, not blockade of gasdermin pore formation or pyroptotic death. For pharmacological interpretation, cytokine reduction, membrane protection, and photoreceptor rescue should therefore be reported as distinct outcomes rather than treated as interchangeable evidence of anti-pyroptotic activity.

Direct GSDMD pore inhibition is attractive because it acts downstream of several inflammasomes. Disulfiram blocks pore formation through cysteine-reactive chemistry, dimethyl fumarate inactivates GSDMD through succination, and necrosulfonamide-related compounds interfere with pore activity (Rathkey et al., 2018; Hu et al., 2020; Humphries et al., 2020). In TREM2-deficient rd10 retina, disulfiram reduced GSDMD oligomerization and photoreceptor loss, providing rare retinal target-engagement evidence (Wang et al., 2026). However, this sensitized genetic context does not establish efficacy in unmodified rd10 disease, and none of these compounds yet has a validated ocular exposure-response relationship, retina-selective target engagement, or gasdermin-specific safety margin. Their electrophilic or pleiotropic chemistry also complicates attribution of benefit to GSDMD alone.

GSDME is a less mature drug target. No selective GSDME pore inhibitor has been validated in retinal tissue, and pharmacological studies mainly act upstream through caspase-3 or oxidative stress. Broad caspase-3 inhibition may preserve membranes but could also delay removal of irreversibly damaged cells. Sustained GSDME suppression likewise requires safety analysis because GSDME contributes to stress-induced cell elimination and chemotherapy-associated lysis in some settings (Wang et al., 2017). A credible GSDME program therefore needs chemical selectivity, retinal target engagement, and evidence that structural rescue is accompanied by preserved visual function rather than prolonged survival of dysfunctional photoreceptors.

Redox- and JNK-directed interventions offer an indirect way to reduce the photoreceptor-intrinsic amplification loop. N-acetylcysteine decreased caspase-3/GSDME activation in the atRAL paradigm, MitoTEMPO reduced mitochondrial oxidative and ferroptotic injury, and JNK inhibition attenuated atRAL-associated photoreceptor ferroptosis (Cai et al., 2022; Yang et al., 2025a; b). These findings are pharmacologically relevant because mitochondrial redox stress may be more tractable than GSDME itself. Nevertheless, antioxidant or kinase benefit cannot be used as evidence of gasdermin engagement unless active-fragment abundance, pore formation, or a Gsdme-dependent treatment interaction is demonstrated.

Across all target classes, five translational gates should be reported. First, target engagement should include the active fragment, oligomer, or pore rather than total protein alone. Second, ocular exposure should link free retinal drug concentration to the effective range. Third, cell specificity should distinguish microglia/macrophages from rods, cones, retinal pigment epithelium, and Müller glia. Fourth, delivery feasibility should be assessed for clinically realistic intravitreal, subretinal, or systemic dosing without assuming that an experimental route is scalable. Fifth, safety should address innate immune competence, membrane repair, tumor surveillance, and treatment duration. Stage is an additional design variable: early myeloid GSDMD suppression may limit inflammatory spread, whereas established photoreceptor injury may require redox or GSDME-directed intervention.

## 6 Discussion and future directions

Three priorities would make the evidence more transferable. First, studies should resolve cellular ownership across disease stages using active-fragment antibodies combined with photoreceptor and myeloid markers, ideally supported by single-cell or spatial profiling. Second, executor dependency should be demonstrated through acute knockdown or conditional deletion followed by rescue with wild-type or cleavage-resistant gasdermin. Third, lytic readouts should be integrated with apoptosis and ferroptosis measurements in the same experiment. A fall in GSDME-N accompanied by preserved mitochondrial function, reduced lipid peroxidation, and improved electroretinography would be more informative than a single protein change.

Inherited retinal degeneration remains the critical next test. P23H and caspase-1 studies establish an inflammasome-sensitive phenotype, while the TREM2-deficient rd10 study now provides direct GSDMD intervention evidence in a sensitized myeloid-inflammatory context (Wang et al., 2026). What remains unresolved is whether GSDMD is required during standard rd10 progression and whether GSDME becomes necessary in rods or cones at defined stages. Comparing rod-dominant and cone-dominant phases with cell-restricted perturbation could reveal whether the two-compartment circuit is a mutation-independent convergence mechanism or a model-specific response.

Extra-retinal evidence that GSDME is p53 responsive and that SIRT1 can deacetylate p53 suggests a possible SIRT1-p53-GSDME modifier axis; however, it should remain a future hypothesis until retinal p53 occupancy, pathway-selective rescue, and GSDME dependency are demonstrated (Masuda et al., 2006; Vaziri et al., 2001; Kadono et al., 2023).

Human translation will require retinal organoids or explants, donor tissue, and longitudinal aqueous or vitreous biomarkers that distinguish executor activation from general inflammation. Biomarker development should prioritize cell-associated GSDMD-N or GSDME-N, pore-linked release products, and pharmacodynamic changes after exposure, while recognizing that soluble cytokines alone cannot identify the executing compartment. These steps would allow early-phase studies to test whether a pharmacologically reachable retinal gasdermin state exists before efficacy trials are attempted.

## 7 Conclusion

Current evidence supports a provisional division of labor: GSDMD predominantly amplifies microglial/macrophage inflammation, whereas GSDME can mediate photoreceptor-intrinsic lytic amplification in the atRAL-clearance paradigm and is associated with greater ferroptotic susceptibility. This framework is more precise than assigning every NLRP3 signal to photoreceptor pyroptosis, but it is not yet universal across inherited retinal degenerations. The most credible pharmacological programs will match executor, cell, disease stage, and delivery route while demonstrating retinal target engagement and functional rescue.
